# Clinical characteristics and prognostic factors analysis in patients with post-neurosurgical intracranial infection caused by *Acinetobacter baumannii*

**DOI:** 10.1186/s12879-025-11597-9

**Published:** 2025-10-09

**Authors:** Bingxin Tian, Huangmin Bao, Xin Yang, Runli Yu, Min Lyu, Wen Liu, Xiaoya Huang, Qin Ding

**Affiliations:** 1 Department of Infectious Disease and Hepatic Disease, The Affiliated Hospital of Xuzhou Medical University, No. 9 Kunpeng North Road, Xuzhou, Jiangsu Province 221006 China; 2 The First Clinical College of Xuzhou Medical University, Xuzhou, Jiangsu Province China

**Keywords:** *Acinetobacter baumannii*, Antimicrobial resistance, Intracranial infection, Prognosis

## Abstract

**Background:**

*Acinetobacter baumannii* intracranial infection is a significant problem affecting the prognosis of neurosurgical patients. This study aimed to analyze the clinical characteristics and identify prognostic factors for in-hospital mortality in patients with post-neurosurgical *A. baumannii* intracranial infections.

**Methods:**

We retrospectively analyzed clinical data from 109 patients diagnosed with *A. baumannii* intracranial infection following neurosurgery between January 2015 and December 2022. A nomogram predicting in-hospital mortality was developed using logistic regression and validated through receiver operating characteristic(ROC) analysis.

**Results:**

A total of 109 patients were included in this study, with an in-hospital mortality rate of 58.7%. Among the *A. baumannii* strains isolated from cerebrospinal fluid(CSF) cultures, 95.4% exhibited carbapenem resistance. Multivariate analysis identified CSF protein level (OR = 1.132, 95% CI = 1.039–1.233), *A. baumannii* bloodstream infection (OR = 8.9, 95% CI = 1.536–51.559), length of stay(OR = 0.946, 95% CI = 0.919–0.974), sex(male)(OR = 0.263, 95% CI = 0.077–0.894), platelet count (OR = 0.996, 95% CI = 0.992–0.999), and albumin level (OR = 0.896, 95% CI = 0.818–0.982) as independent factors associated with in-hospital mortality. A nomogram incorporating these factors achieved an area under the curve(AUC) of 0.879 (95% CI = 0.806–0.952).

**Conclusion:**

Post-neurosurgical intracranial infection with *A. baumannii* is associated with a high in-hospital mortality rate. Our study identified several independent prognostic factors, including CSF protein level, *A. baumannii* bloodstream infection, length of stay, sex(male), platelet count, and albumin level. These findings highlight the need for early identification and targeted interventions to improve patient outcomes.

## Background

Intracranial infections following neurosurgery are a significant cause of morbidity and mortality. According to the China Antimicrobial Surveillance Network, the proportion of Gram-negative bacteria in the cerebrospinal fluid(CSF) has been increasing annually, with *Acinetobacter baumannii* being the most prevalent [1].

*A. baumannii* is a non-fermenting Gram-negative bacillus that possesses a range of virulence factors, including membrane pore proteins, periplasmic membrane polysaccharides, phospholipases, outer membrane vesicles, and iron uptake, among others [2]. It exhibits significant natural and acquired resistance through mechanisms such as the production of drug-resistant enzymes, the alteration of drug targets, the efflux pumping system, the alteration of membrane permeability, and the production of biofilms, demonstrating robust resistance to environmental stressors and antimicrobial agents [3, 4]. *Multidrug-resistant A. baumannii* has shown an epidemic trend worldwide, posing severe challenges in managing central nervous system(CNS) infections [5, 6]. Additionally, the blood-brain barrier(BBB) further complicates treatment by limiting the penetration of most antimicrobial agents into the CSF, resulting in the concentration of most drugs in CSF is significantly lower than that in serum, and it is difficult for antibacterial drugs to reach the minimum inhibitory concentration(MIC), which limits the choice of therapeutic agents for the treatment of intracranial infections with *A. baumannii*.

Post-neurosurgical intracranial infections caused by *A. baumannii* remain a major challenge in neurosurgical patient management. Previous studies have mainly focused on identifying risk factors for infection [7–9]. Or have reported individual treatment cases in critically ill patients [10–12], while few have investigated factors associated with patient prognosis. A deeper understanding is needed to facilitate the development of effective treatments and enhance patient outcomes. This study aims to investigate the clinical characteristics and prognostic factors associated with this condition, with the goal of providing evidence to support risk stratification and individualized management in this high-risk patient population.

## Materials and methods

### Study population and data collection

**Fig. 1:**
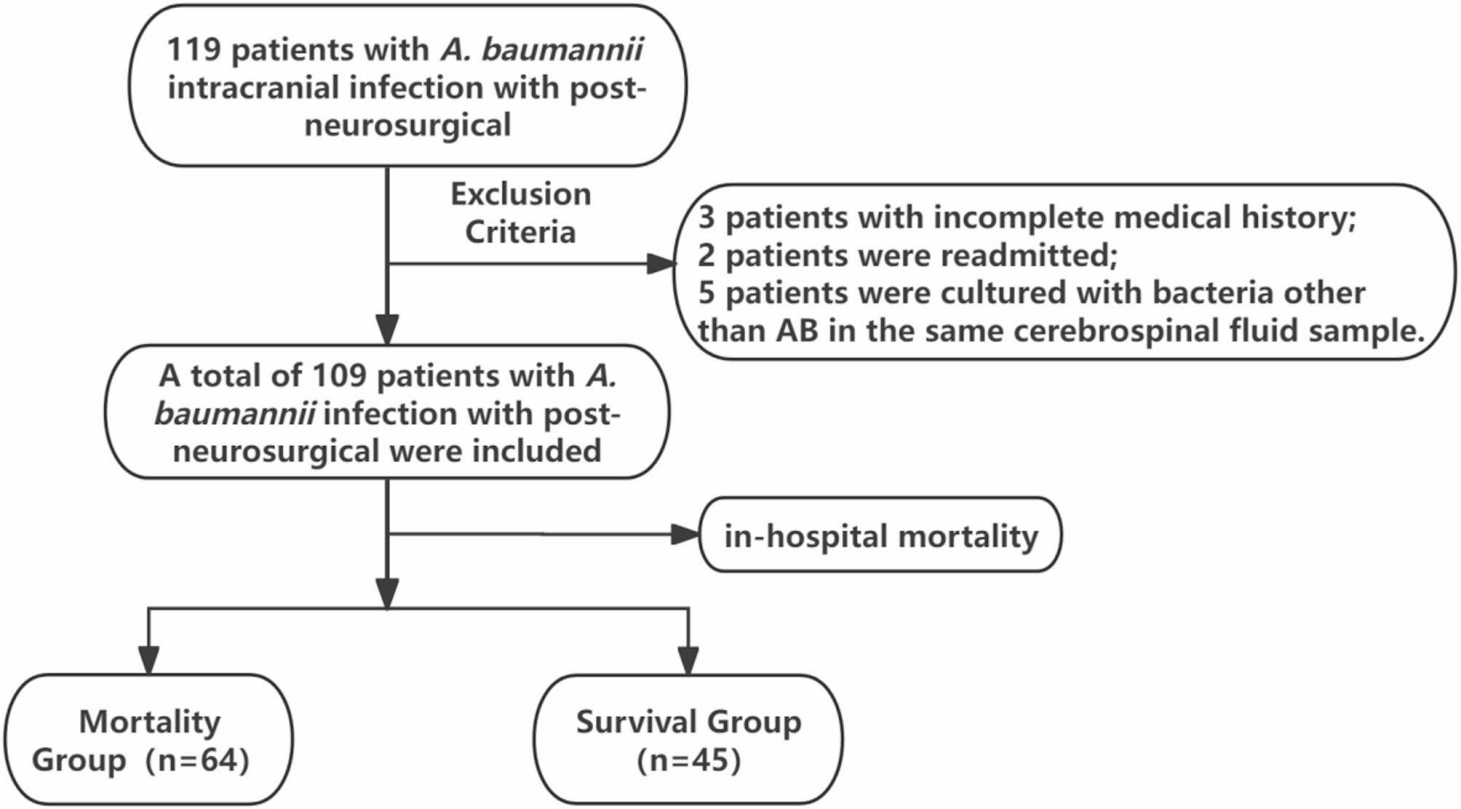
Flow chart

The medical records of patients with post-neurosurgical *A. baumannii* intracranial infections diagnosed at the Affiliated Hospital of Xuzhou Medical University from January 2015 to December 2023 were retrospectively analyzed. In this study, *A. baumannii* intracranial infection was defined according to the 2017 Infectious Diseases Society of America’s Clinical Practice Guidelines for Healthcare-Associated Ventriculitis and Meningitis and the European Society for Clinical Microbiology and Infectious Diseases guidelines: diagnosis and treatment of acute bacterial meningitis [13, 14]. All the included patients met the criteria for laboratory diagnosis, and CSF cultures were positive for *A. baumannii* strains. We collected the following information on patients with *A. baumannii* isolated from CSF: demographics, comorbidities, primary craniocerebral diseases, history of intracranial surgical interventions, symptoms and signs, CSF parameters, laboratory findings, use of medical devices, mechanical ventilation, co-infections, isolation of *A. baumannii* from blood and sputum, presence of septic shock as an indicator of severity, pre-existing or concurrent conditions, treatment, length of hospital stay, and in-hospital mortality as the outcome measure. Hematology and CSF parameters were collected retrospectively on the day of collection of CSF specimens with positive *A. baumannii* cultures. Multiple isolations of *A. baumannii* from CSF were considered a single event, and only data from the initial event were analyzed. Patients under 18 years of age, those with incomplete data, or those who did not undergo neurosurgical procedures were excluded from the analysis. Furthermore, Patients who underwent neurosurgical procedures specifically for the treatment of intracranial infections (e.g., abscess evacuation or debridement) were excluded from this study. The flow chart is shown in Fig. 1. The study was approved by the Ethics Committee of the Affiliated Hospital of Xuzhou Medical University (approval number: XYFY2024 - KL401-01).

### A. baumannii identification and antimicrobial susceptibility

All isolated *A. baumannii* strains were cultured using Columbia blood agar and chocolate agar. Identification of *A. baumannii* at the species level was performed using matrix-assisted laser desorption/ionization time-of-flight mass spectrometry (MALDI-TOF MS) with the autoflex maX system (Bruker Daltonics, Bremen, Germany) [15]. Antimicrobial susceptibility testing and minimum inhibitory concentration (MIC) determination were carried out according to the Clinical and Laboratory Standards Institute (CLSI) guidelines for antimicrobial susceptibility testing (M100, 31 st edition) [16]. The antimicrobial agents were mixed in a gradient agar medium, inoculated with the bacterial suspension, incubated, and the lowest concentration inhibiting visible growth was recorded.

Multidrug-resistant(MDR) is defined as being insensitive to ≥ 1 agent in ≥ 3 antimicrobial categories(Fluoroquinolones, Cephalosporins, β-Lactamase Inhibitors, Carbapenems, Aminoglycosides) [17–19]. The MIC breakpoint of carbapenem-resistant *A. baumannii* is that the MIC of donipenem, imipenem, and meropenem is greater than or equal to 8 ug/mL [20].

### Management of intracranial infection

Management of patients with post-neurosurgical intracranial infections was conducted in accordance with the 2021 Chinese Expert Consensus on the Diagnosis and Treatment of Central Nervous System Infections in Neurosurgery, and the 2017 Infectious Diseases Society of America (IDSA) Clinical Practice Guidelines for Healthcare-Associated Ventriculitis and Meningitis.

Once intracranial infection was suspected, empirical antimicrobial therapy was initiated promptly. According to guideline recommendations, vancomycin was administered in combination with either an anti-pseudomonal cephalosporin (e.g., ceftazidime or cefepime) or a carbapenem (e.g., meropenem), based on local resistance patterns. Antimicrobial regimens were subsequently adjusted according to CSF culture and susceptibility testing results to ensure targeted therapy. For patients with external ventricular drainage (EVD), catheter removal or replacement was performed when feasible, especially in cases of confirmed or suspected device-associated infection. In patients who developed hydrocephalus secondary to infection, ventriculoperitoneal shunting (VPS) was considered following infection control and clinical stabilization. Surgical wounds were managed with routine sterile dressing changes and were closely monitored for signs of local infection. If necessary, wound debridement was performed. Supportive care included intracranial pressure management, fluid and electrolyte balance, nutritional support, and organ function monitoring. Critically ill patients were treated in the intensive care unit (ICU) when required. Multidisciplinary collaboration among neurosurgeons, infectious disease specialists, critical care teams, and microbiologists was implemented throughout the course of care to ensure individualized and dynamic treatment plans.

### Statistical methods

For continuous variables, normally distributed variables are expressed as the means ± standard deviations and were analyzed by two independent samples t-tests, and non-normally distributed variables are expressed as medians(25–75% quartiles), and differences were analyzed via the Mann-Whitney U test. Categorical variables are expressed as percentages (%) and were analyzed using a chi-square test or Fisher’s exact test. Univariates associated with death at *P* < 0.2 were included in the multivariate logistic regression analysis to identify independent prognostic factors for mortality, calculating odds ratios (ORs) and 95% confidence intervals (CIs). The ORs represent the likelihood of mortality associated with each prognostic factor, with values greater than 1 indicating an increased risk and values less than 1 indicating a decreased risk. The 95% CIs provide a range within which the true OR is likely to fall, and a CI that does not include 1 indicates statistical significance. This approach allows us to quantify the impact of each factor on patient outcomes and prioritize interventions accordingly. A nomogram was constructed using all the independent prognostic factors [21]. The predictive accuracy was assessed via the receiver operating characteristic (ROC) curve and area under the curve (AUC), and calibration curves were used to evaluate the predictive accuracy. *P* < 0.05 (two-sided) was considered statistically significant in all analyses. SPSS 27.0 and R version 4.4.1 software were used for statistical analysis.

## Results

### Demographics, underlying conditions, and craniocerebral disease of patients with post-neurosurgical A. baumannii intracranial infection

**Fig. 2:**
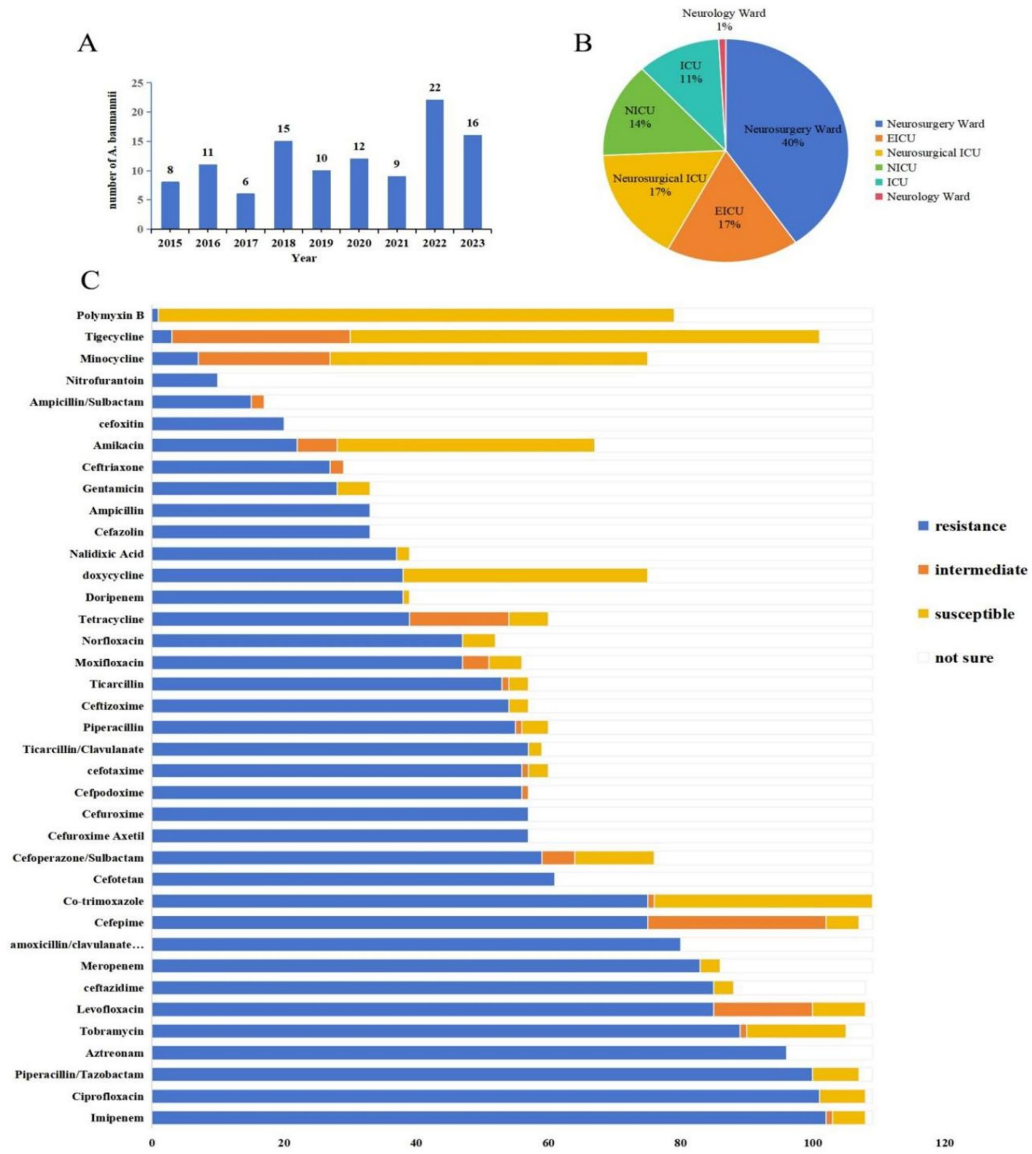
Distribution and antimicrobial resistance of *A. baumannii* strains. **A** The annual distribution of intracranial *A. baumannii* infections following neurosurgical procedures. **B** The departmental distribution of *A. baumannii* strains isolated from cerebrospinal fluid cultures. **C** Resistance rates and susceptibility rates of *A. baumannii* to various antibiotics. *A. baumannii* = *Acinetobacter baumannii*; ICU = Intensive Care Unit; EICU = Emergency Intensive Care Unit; NICU = Neurosurgical intensive Care Unit

A total of 109 patients diagnosed with post-neurosurgical *A. baumannii* intracranial infection were included in this study, with 64(58.7%) in the death group and 45 in the survival group based on prognosis. There were 85(78.0%) males and 24(22.0%) females, with a mean age of 52.68 ± 12.15 years, significantly higher in the death group compared to the survival group. The annual distribution of *A. baumannii* strains in CSF samples is illustrated in Fig. 2A, with the neurosurgery ward exhibiting the highest proportion of isolates. The remaining isolates were found in various intensive care units(ICUs), and a case was identified in the neurology ward, as shown in Fig. 2B. Hypertension and diabetes mellitus are common underlying diseases; however, there was no significant difference between the death and survival groups. A total of 109 patients had various Primary craniocerebral diseases, including cerebral hemorrhage, head trauma, aneurysm, intracranial benign and malignant tumors, CSF leakage, and cerebral infarction, All patients received craniotomy intervention. The baseline results of the two groups are shown in Table 1.

**Table 1:** Baseline characteristics of patients with intracranial infection due to *A. baumannii* post-neurosurgical

	Total(*n* = 109)	Mortality Group(*n* = 64)	Survival Group(*n* = 45)	*P*-value
Demographic Characteristics				
Sex (Male) (%)	85(78.0)	47(73.4)	38(84.4)	0.172
Age (years)	52.68 ± 12.15	54.88 ± 12.35	49.56 ± 11.26	0.024^*^
Comorbidities (%)				
Hypertension	64(58.7)	38(59.4)	26(57.8)	0.868
Diabetes Mellitus	13(11.9)	9(11.4)	4(8.9)	0.412
Primary craniocerebral diseases (%)				
Intracerebral Hemorrhage	104(95.4)	63(98.4)	41(91.1)	0.072
Head Trauma	25(22.9)	14(21.9)	11(24.4)	0.753
Aneurysm	15(13.8)	6(9.4)	9(20.0)	0.113
Intracranial Benign Tumor	3(2.8)	2(3.1)	1(2.2)	0.777
Intracranial Malignant Tumor	1(0.9)	0(0.0)	1(2.2)	0.859
CSF Leak	10(9.2)	7(10.9)	3(6.7)	0.672
Cerebral Infarction	37(33.9)	19(29.7)	18(40.0)	0.263

*CSF* Cerebrospinal fluid

*P < 0.05

### Clinical characteristics and CSF parameters of patients with Post-neurosurgical A. baumannii intracranial infection

Symptomatically, the most common presentation of the patients was fever(93.6%), followed by Glasgow Coma Scale(GCS) score ≤ 8 points (80.7%), with a GCS score ≤ 8 points being significantly higher in the death group than in the survival group(*P* = 0.033). Other common symptoms, such as headache, nausea, and vomiting, did not differ significantly between the two groups.

Regarding CSF indexes, CSF leukocytes (*P* = 0.037) and CSF multinucleated cells (*P* = 0.031) were higher in patients in the death group compared to the survival group. In addition, CSF erythrocytes (7.5*10^9/L vs. 1*10^9/L, *P* = 0.001) and CSF protein level (9.72 g/L vs. 4.5 g/L, *P* < 0.001) were significantly higher in the death group than in the survival group.

Hematologic indices revealed that aspartate transaminase(AST) (*P* = 0.005) was higher in the death group than in the survival group, with a statistically significant difference. Moreover, the platelet count (*P* = 0.001), platelet crit(PCT) (*P* = 0.003), cholinesterase(CHE) (*P* = 0.022), and albumin(ALB) level (*P* = 0.001) were lower in patients in the death group than in the survival group, with statistically significant differences. The results are shown in Table 2.

**Table 2:** Clinical characteristics of patients with intracranial infection due to *A. baumannii* post-neurosurgical

	Total (*n* = 109)	Mortality Group (*n* = 64)	Survival Group (*n* = 45)	*P*-value
Symptomatology (%)				
Fever (T ≥ 38.5 °C)	102(93.6)	59(92.2)	43(95.6)	0.757
GCS ≤ 8 points	88(80.7)	56(87.5)	32(71.1)	0.033^*^
Headache	34(31.2)	20(31.3)	14(31.1)	0.988
Nausea	58(53.2)	33(51.6)	25(55.6)	0.681
Vomiting	52(47.7)	30(46.9)	22(48.9)	0.836
Positive Meningeal Irritation	19(17.4)	9(14.1)	10(22.2)	0.269
Unequal pupils	29(26.6)	18(28.1)	11(24.4)	0.669
CSF Parameters				
RBC (10^9/L)	4(1.0, 18.5)	7.5(2.0, 33.0)	1(0.0, 7.0)	0.001^*^
WBC (10^6/L)	4603(1084.5, 17750.5)	6464(1367.0, 22609.8)	2617(417.5, 11421.5)	0.037^*^
Polymorphonuclear cells(10^6/L)	3825(898.0, 16888.5)	5850.5(1276.8, 21588.0)	2184(284.0, 10712.5)	0.031^*^
Glucose (mmol/L)	1.11(1.11, 2.34)	1.11(1.11, 2.97)	1.11(1.11, 2.01)	0.132
Chloride (mmol/L)	111.9(106.5, 119.2)	114.65(106.5, 120.4)	109.6(105.7, 113.7)	0.204
Protein (g/L)	6.82(3.3, 14.1)	9.72(4.9, 15.0)	4.5(2.4, 8.3)	<0.001^*^
Hematological Parameters				
WBC(10^9/L)	12.9(9.65, 17.35)	12.55(9.08, 17.25)	13.6(10.15, 17.50)	0.582
PLT(10^9/L)	296(208.5, 418.5)	251(184.75, 381.25)	348(270.5, 439.0)	0.001^*^
CRP(mg/L)	94.461(48.1, 110.3)	93.4184(68.78, 110.80)	83.6(36.6, 109.2)	0.101
PCT(%)	0.3(0.23, 0.39)	0.26(0.19, 0.35)	0.33(0.26, 0.44)	0.003^*^
AST (U/L)	37(19, 72)	51.5(22.0, 88.5)	30(15.5, 49.0)	0.005^*^
CHE (U/L)	4617.16 ± 1613.13	4321.64 ± 1675.51	5037.44 ± 1589.16	0.022^*^
ALB (g/L)	32.4(29, 35.65)	31.4(28.275, 34.75)	34(31.6, 38.1)	0.001^*^
CSF/Blood Ratios				
Chloride Ratio	1.0997 ± 0.0860	1.0934 ± 0.0917	1.1087 ± 0.0773	0.363
Glucose Ratio	0.1511(0.1012, 0.2194)	0.1382(0.0985, 0.1903)	0.1756(0.1044, 0.2863)	0.105

*GCS* Glasgow Coma Scale, *CSF* Cerebrospinal fluid, *RBC* Erythrocyte, *WBC* Leukocyte, *PLT* Blood platelet, *CRP* C-Reactive Protein, *PCT* Plateletcrit, *AST* Glutamic-pyruvic transaminase, *CHE* Choline esterase, *ALB* Albumin

*P < 0.05

In 34 patients with intracranial *A. baumannii* infection, additional bacterial species were isolated from CSF samples during hospitalization, indicating polymicrobial infection in these cases. The most frequently co-isolated organism was *Klebsiella pneumoniae*. In terms of pre-existing or concurrent conditions, hydrocephalus was common(46.8%), while infectious shock was more significant in the death group (*P* = 0.02).The results are shown in Table 3.

**Table 3:** Invasive procedures, co-infections, and preexisting or concurrent conditions of patients with post-neurosurgical *A. baumannii* intracranial infections

	Total (*n* = 109)	Mortality Group (*n* = 64)	Survival Group (*n* = 45)	*P*-value
Invasive procedures & co-infections (%)				
Invasive Ventilation	103(94.5)	62(96.9)	41(91.1)	0.383
Pulmonary Infection	103(94.5)	62(96.9)	41(91.1)	0.383
CSF Culture with Other Bacteria	34(31.2)	19(29.7)	15(33.3)	0.686
* A. baumannii* bloodstream infection	16(14.7)	13(20.3)	3(6.7)	0.088
Sputum Culture Positive for *AB*	81(74.3)	50(78.1)	31(68.9)	0.277
Nasogastric Tube	107(98.2)	64(100.0)	43(95.6)	0.168
Urethral Catheter	108(99.1)	64(100.0)	44(97.8)	0.413
Central Venous Catheter	72(66.1)	44(68.8)	28(62.2)	0.479
Pre-existing or concurrent conditions(%)				
Hydrocephalus	51(46.8)	30(46.9)	21(46.7)	0.983
Epilepsy	8(7.3)	4(6.3)	4(8.9)	0.883
Septic Shock	8(7.3)	8(12.5)	0(0.0)	0.020^*^
Brain Herniation	5(4.6)	2(3.1)	3(6.7)	0.685

*CSF* Cerebrospinal fluid, *AB/A. baumannii*/*Acinetobacter baumannii*

*P < 0.05

### Treatment and outcomes of patients with post-neurosurgical A. baumannii intracranial infection

Regarding treatment, the vast majority of patients received an empirical choice of antibiotics (99.1%), including 64 (100.0%) in the death group and 44 (97.8%) in the survival group. Thirty-eight (34.9%) patients received intrathecal injection, 24 (37.5%) in the death group and 14 (31.1%) in the survival group. There was no significant difference between the two groups (*P* = 0.491). After antimicrobial sensitivity results, 79 patients received sensitive antibiotics, and there was no significant difference between the two groups (*P* = 0.546). High-dose application of Steroids was more common in the death group (54.7% vs. 40%). VPS was more common in the surviving group(26.7% vs.7.8%, *P* = 0.008). The length of stay was significantly longer in the survivor group (*P* < 0.001). The results are shown in Table 4.

**Table 4:** Treatment and outcomes of patients with post-neurosurgical *A. baumannii* intracranial infection

	Total (*n* = 109)	Mortality Group (*n* = 64)	Survival Group (*n* = 45)	*P*-value
Treatment(%)				
Empirical antibiotic use	108(99.1)	64(100)	44(97.8)	0.413
Drug-sensitive antibiotics	79(72.5)	45(70.3)	34(75.6)	0.546
Intrathecal Injection of antibiotics	38(34.9)	24(37.5)	14(31.1)	0.491
High-dose steroid use	53(48.6)	35(54.7)	18(40)	0.131
Surgical interventions (%)				
EVD	108(99.1)	64(100.0)	44(97.8)	0.413
VPS	17(15.6)	5(7.8)	12(26.7)	0.008^*^
LD	100(91.7)	57(89.1)	43(95.6)	0.390
Hospital Stay(%)				
Hospitalization duration	28(18.5, 43)	23.5(14.00, 35.75)	37(24.5, 53.5)	<0.001^*^
ICU stay	13(6, 24.5)	13.5(7.00, 24.75)	9(5, 27)	0.205

*ICU* Intensive Care Unit, *mRS* Modified Rankin scale, *EVD* External Ventricular Drainage, *VPS* Ventriculoperitoneal Shunt, *LD* Lumbar drainage

*P < 0.05

As shown in Table 5, the multivariate analysis on prognostic factors for in-hospital mortality showed that CSF protein level (OR = 1.132, 95% CI = 1.039–1.233, *P* = 0.005), *A. baumannii* bloodstream infection (OR = 8.9, 95% CI = 1.536–51.559, *P* = 0.015), length of stay(OR = 0.946, 95% CI = 0.919−0.974, *P* < 0.001), sex (male)(OR = 0.263, 95% CI = 0.077–0.894, *P* = 0.032), platelet count (OR = 0.996, 95% CI = 0.992–0.999, *P* = 0.013), and albumin level (OR = 0.896, 95% CI = 0.818–0.982, *P* = 0.019) were identified as independent predictors of in-hospital mortality.

**Table 5:** Multivariable logistic regression analysis of mortality predictors in intracranial infection due to *A. baumannii*

	β	SE	Wald	OR	95%OR (Lower–Upper)	*P*	1/VIF	VIF
CSF Protein level	0.124	0.044	8.045	1.132	1.039–1.233	0.005^*^	0.966	1.035
*A. baumannii* bloodstream infection	2.186	0.896	5.948	8.9	1.536–51.559	0.015^*^	0.972	1.028
Length of Stay	−0.056	0.015	13.824	0.946	0.919–0.974	<0.001^*^	0.996	1.004
Sex (Male)	−1.337	0.625	4.578	0.263	0.077–0.894	0.032^*^	0.979	1.021
Platelet count	−0.005	0.002	6.210	0.996	0.992–0.999	0.013^*^	0.944	1.059
Albumin level	−0.11	0.047	5.534	0.896	0.818–0.982	0.019^*^	0.938	1.066

*CSF* Cerebrospinal fluid, *A. baumannii* *Acinetobacter baumannii*

*P < 0.05

### A model for predicting In-Hospital mortality using prognostic factors

**Fig. 3:**
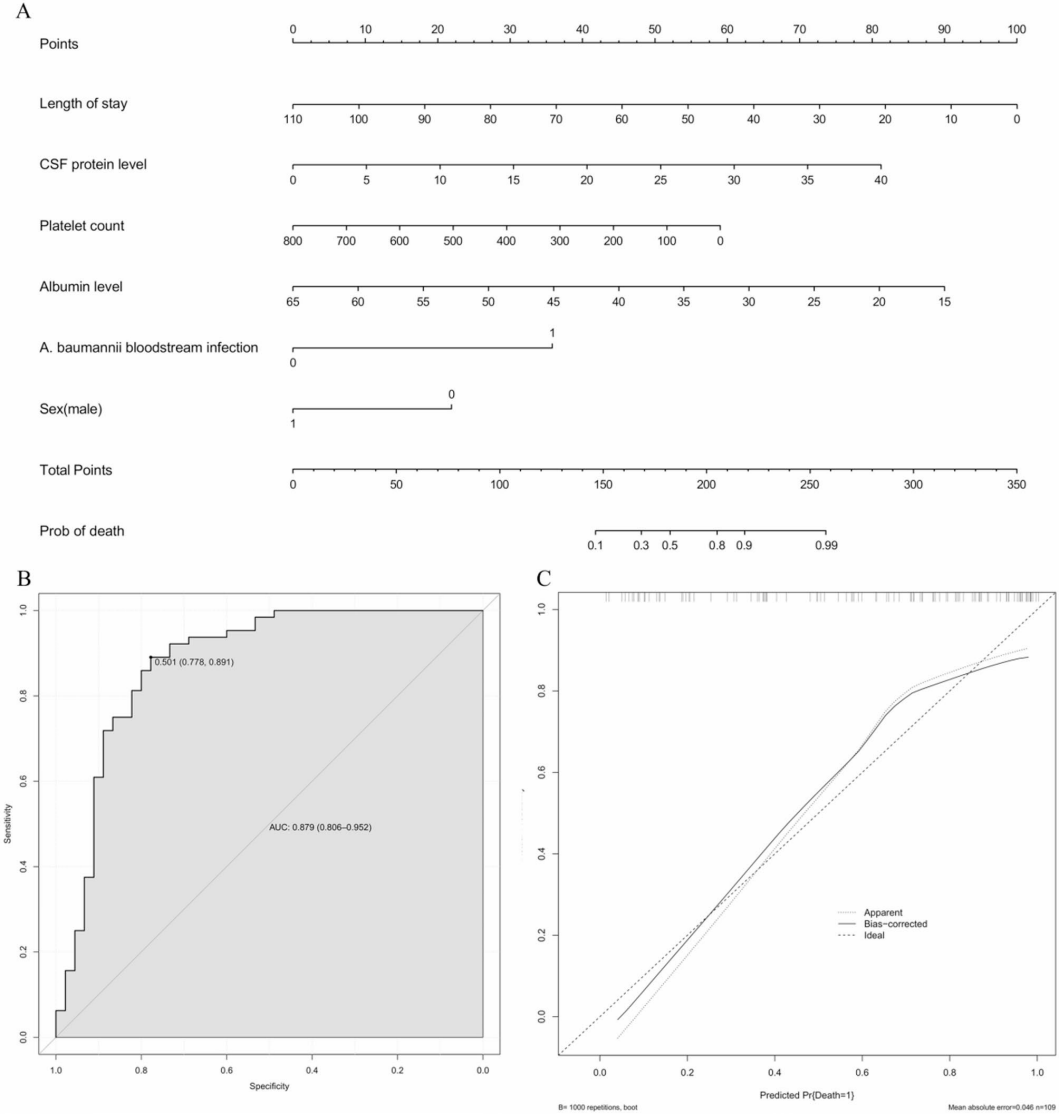
A nomogram for patients with post-neurosurgical *A. baumannii* intracranial infection. **A** Nomogram for predicting in-hospital mortality rates in patients. **B** ROC curve of the nomogram for predicting death in patients. The AUC of the ROC curve was 0.879 (95% CI, 0.806–0.952). **C** Calibration curves for death in patients. ROC, receiver operating characteristic; AUC, area under the curve

A nomogram was constructed using the independent prognostic factors identified above, with each variable assigned a score based on its value. The total score was calculated by summing these individual scores to predict patient mortality risk (Fig. 3A). The evaluation of the constructed prognostic model indicated an AUC of 0.879 (95% CI = 0.806–0.952) (Fig. 3B), and the calibration plot demonstrated a good fit for the logistic regression model(Fig. 3C).

### Antimicrobial susceptibility of A. baumannii

The carbapenem resistance rate of the isolated *A. baumannii* strains was high(95.4%), the multidrug resistance rate was high(90%), and the extensively drug-resistance rate was low (24.8%). There was no statistical significance between the death group and the survival group (*P* > 0.05). According to the antimicrobial sensitivity test results, *A. baumannii* has a high resistance rate to common antibiotics. The resistance rate of cefotaxime, ceftazidime, amoxicillin/clavulanate potassium, and imipenem was > 90%. The resistance rates of *A. baumannii* to tigecycline and polymyxin are relatively low, at 2.97% and 1.27%, respectively. The results of the antimicrobial drug resistance tests are presented in Tables 6 and 7.

**Table 6:** Antibiotic resistance testing of *A. baumannii* strains isolated from the mortality and survival groups

	Total (*n* = 109)	Mortality Group (*n* = 64)	Survival Group (*n* = 45)	*P*-value
Antimicrobial Resistance Rates (%)				
Carbapenem-resistant *A. baumannii*	104(95.4)	61(95.3)	43(95.6)	1.000
Multidrug-resistant *A. baumannii*	98(90.0)	57(89.1)	41(91.1)	0.979
Extensively drug-resistant *A. baumannii*	27(24.8)	13(20.3)	14(31.1)	0.198

*A. baumannii* *Acinetobacter baumannii*

*P < 0.05

**Table 7:** Antibiotic drug resistance testing of *A. baumannii* strains isolated from CSF cultures

	S[*n*(%)]	I[*n*(%)]	*R*[*n*(%)]
Fluoroquinolones(%)			
Levofloxacin	8(7.41)	15(13.89)	85(78.70)
Moxifloxacin	5(8.93)	4(7.14)	47(83.93)
Cephalosporins(%)			
Cefepime	5(4.67)	27(25.23)	75(70.09)
Cefotaxime	3(5.00)	1(1.67)	56(93.33)
Ceftazidime	3(3.37)	0(0.00)	86(96.63)
β-Lactamase Inhibitors(%)			
Cefoperazone/Sulbactam	12(15.79)	5(6.58)	59(77.63)
Amoxicillin/Clavulanate	0(0.00)	0(0.00)	77(100.00)
Carbapenems(%)			
Imipenem	5(4.63)	1(0.93)	102(94.44)
Meropenem	3(3.49)	0(0.00)	83(96.51)
Aminoglycosides(%)			
Amikacin	39(58.21)	6(8.96)	22(32.84)
Gentamicin	5(15.15)	0(0.00)	28(84.85)
Tobramycin	15(14.29)	1(0.95)	89(84.76)
Others(%)			
Tigecycline	71(70.30)	27(26.73)	3(2.97)
Polymyxin B	78(98.73)	0(0.00)	1(1.27)
Trimethoprim/Sulfamethoxazole	33(30.28)	1(0.92)	75(68.81)

*S Susceptible, I Intermediate, R Resistance*

## Discussion

Intracranial infection with *A. baumannii* after neurosurgery imposes a significant burden on the healthcare system. Disruption of the BBB in postoperative patients increases susceptibility to pathogenic microorganisms, thereby elevating the risk of intracranial infections and poor prognosis. The increasing antimicrobial resistance of *A. baumannii* complicates treatment. According to the relevant literature, the mortality rate of *A. baumannii* intracranial infection ranges from 15 to 71%, and this study reported an in-hospital mortality rate of 58.7% [6], which is consistent with previous reports. Multivariate logistic regression analysis identified sex(male), CSF protein level, platelet count, *A. baumannii* bloodstream infection, length of stay, and albumin level as independent predictors of prognosis. Nomograms were constructed based on the results of multiple logistic regression analysis, achieving an AUC of 0.879.

### Predictors of in-hospital mortality

Our findings showed that longer hospital stay was statistically associated with a lower risk of in-hospital mortality. As this is a retrospective study, this result may reflect survivor bias, in which patients who survive longer naturally accumulate more hospital days. In addition, prolonged hospitalization may also indicate that the patient responded to treatment over time and was stable enough to continue receiving care. Some patients required extended hospital stays to complete a full course of antibiotic therapy after repeated positive CSF cultures. According to the Infectious Diseases Society of America Guidelines on Meningitis and Ventriculitis, the recommended duration of antibiotics for patients with gram-negative bacilli intracranial infections is 10−14 days, with continuation of treatment for another 10−14 days if multiple cultures remain positive after treatment [13]. According to De Bonis P et al.., the time to convert CSF cultures to negative after treatment was 21 days [22]. Therefore, length of stay may serve as a proxy indicator of clinical stability and treatment continuity among survivors.

Elevated CSF protein levels were strongly associated with in-hospital mortality, which likely reflects BBB disruption, facilitating bacterial invasion and amplifying neuroinflammation. The presence of proteins in CSF may significantly influence the expression of various *A. baumannii* genes, which indirectly affects bacterial motility, biofilm formation, efflux pumps, metabolism, transcriptional regulation, and antibiotic resistance (e.g., by affecting the down-regulation of the outer membrane protein *CarO* and up-regulation of the expression of β-lactamases, increasing the resistance of *A. baumannii* to β-lactam antibiotics) [23, 24]. This may affect the speed of recovery and prognosis of patients.

In our study, lower platelet count was independently associated with increased mortality risk, although the median platelet level in the death group remained within the normal range. This association may reflect platelet consumption in patients with more severe systemic inflammation or sepsis, consistent with prior observations in critically ill populations. Platelets are known to play crucial roles in neuroinflammation and BBB integrity, and platelet-derived bioactive molecules possess anti-inflammatory, neuroprotective, and antioxidant properties, potentially improving the prognosis of patients with cerebral hemorrhage. On the one hand, platelets may promote neurogenesis by carrying and releasing neurotrophic factors (such as *VEGF*, *IGF-1*, *FGF-2*, etc.). On the other hand, platelets have dual effects on BBB integrity in brain pathology: first, vascular integrity is likely protected by the formation of thrombosis. Then, BBB is likely destroyed by platelets releasing various factors (*PDGF-AA*, *PDGF-CC*, etc.) [25, 26]. Although these mechanisms were not directly evaluated in our study, the results suggest that clinical dynamic platelet monitoring has value for disease progression.

In our study, the male gender appeared to be associated with a lower mortality rate, which contrasts with findings from previous studies on bacterial meningitis and nosocomial CNS infections, where male patients often exhibited worse outcomes. For instance, Hsieh [27] reported that male was more frequently associated with nosocomial infections and adverse prognoses, particularly in the setting of neurosurgical procedures and traumatic brain injury, and a higher prevalence of smoking and drinking, which may contribute to poorer immune function in males [4]. The discrepancy observed in our study may be attributable to an imbalance in sex distribution between the survival and death groups, as well as the relatively small sample size. Further investigation with a larger cohort is warranted to confirm whether this observation represents a true protective association or a statistical artifact.

In our study, lower serum albumin levels were independently associated with increased in-hospital mortality. Hypoalbuminemia may reflect underlying disease severity, systemic inflammation, or malnutrition, all of which are common in critically ill neurosurgical patients.Previous studies have demonstrated that for critically ill patients, every 10 g/L decrease in protein will increase the hospital mortality rate by 137% [28]. Low protein levels also impact the pharmacokinetics of antibiotics, and improving a patient’s hypoproteinemic state is more likely to enhance prognosis and aid in the recovery of neurologic function [29]. The value of interventions targeting hypoalbuminemia in improving survival among these patients requires further investigation.

*A. baumannii* bloodstream infection was identified as an independent risk factor for in-hospital mortality in our study. This association may reflect the overall bacterial burden and systemic inflammatory response in critically patients. While sustained bacteremia has been proposed as a potential route for microbial entry into the central nervous system. In the absence of other sources of infection, intracranial infection may also be a source of bacteremia. Sustained (high-grade) bacteremia is thought to be necessary, although not sufficient, for microbial entry into the subarachnoid space [30]. Therefore, although bacteremia is clearly associated with more severe disease, its precise role in the pathophysiology of *A. baumannii* CNS infections warrants further investigation.

Notably, in this study, VPS was much more prevalent in the survivor group than in the death group, which is consistent with the study by Shige Li et al. [7]. Cerebral drainage of CSF can relieve acute intracranial pressure and hydrocephalus, improve the circulation of CSF, avoid the occurrence of cerebral herniation, minimize nerve damage, and reduce the retention time of infectious sources in the brain. *A. baumannii* is a common environmental organism in healthcare settings. The catheter for the VPS, which is placed distally in the abdominal cavity through a subcutaneous channel, is not connected to the external environment, which reduces the risk of retrograde infection.

### Antimicrobial resistance

*A. baumannii* is one of the most severe nosocomial multidrug-resistant pathogens. *A. baumannii* adheres to eukaryotic cells via various virulence factors, such as pili, V-type autotransporter secretion systems (*Ata* and *FhaB/C*), outer membrane proteins (*OmpA*), invasin-like adhesins secreted by the *T2SS*, adhesins, the *TonB* system and the ferrous transport system (*FeoABC*), with *OmpA* being extensively studied [2, 4]. The antimicrobial resistance mechanisms of *A. baumannii* include and so on: (1) Mutations or horizontal transfer of efflux pump-related genes, especially *Ade ABC*, *Ade FGH*, and *Ade IJK* in the efflux system of the resistance-nodulation division family (*RND*). (2) Antibiotic target mutations: for example, when the mutation of penicillin-binding proteins (*PBPs*) occurs, the affinity of antibiotics and PBPs decreases, reducing the effectiveness of antibiotics. (3) A high resistance rate to carbapenems was generally observed, which is also consistent with our study. The production of antibiotic-modifying enzymes (β-lactamase) promotes the formation of multiple drug-resistant strains. β-Lactamase can be divided into A, B, C, and D classes. Among them, the main subtypes of the D class are OXA-23 and OXA-51, which are an important part of antimicrobial drug resistance mechanisms. (4) Decreased permeability associated with biological membrane and outer membrane proteins: The outer membrane of *A. baumannii* contains a variety of outer membrane proteins (*OMPs*), including *OmpA*, *Omp 33–36*, *Omp 22*, etc. *OmpA*, which encodes one of the most abundant porin proteins in the outer membrane of *A. baumannii*, participates in biofilm formation. Understanding the associated virulence factors of *A. baumannii* and their resistance mechanisms could facilitate the discovery of novel therapies. In a study by Chisook Moon et al.., the prognosis of patients with *A. baumannii* intracranial infections was considered to be related to the carbapenem resistance rate [31]. In our study, the carbapenem resistance rate did not affect the prognosis of patients, probably due to our insufficient sample size and our included data 95.4% of *A. baumannii* isolates showed carbapenem resistance.

### Diagnosis

Early diagnosis of intracranial infections caused by *A. baumannii* is essential because of its refractory nature and high mortality. The CSF leukocyte count remains the most efficient and reliable predictor [32]. The gold standard for diagnosis remains CSF culture for pathogens, which typically requires 48–72 h. Polymerase chain reaction (PCR) assays have improved in efficiency and show high specificity, but their expense is limited, and comprehensive pathogen coverage is lacking [33]. Macrogenomic next-generation sequencing (mNGS) is an emerging rapid assay, and the average detection time for mNGS in Ying Liu’s study was 1.4 days [34]. Lei Yuan et al..‘s study revealed that the mNGS method could detect a broader range of pathogens than traditional methods. However, inconsistencies between mNGS results and CSF cultures are common. mNGS results should be analyzed in conjunction with the patient’s clinical symptoms, pathogenicity, and cell and protein levels in the CSF [35]. Some studies have shown that the detection rate of mNGS is higher when the CSF WBC count is > 300*10^6/L, the CSF protein level is > 500 mg/L, or the CSF to blood serum glucose ratio is ≤ 0.3, which may suggest that we tend to use this test in patients with high CSF cell counts [36, 37]. In addition, MNCP-II is a highly effective molecular detection platform that significantly improves diagnostic efficiency and reduces detection times [38].

### Treatment

According to the Infectious Diseases Society of America guideline recommendations, immediate empiric antimicrobial therapy is necessary while awaiting culture results [13], and empiric treatment of intracranial infections includes vancomycin in combination with carbapenems such as meropenem. After cerebrospinal fluid culture for *A. baumannii*, medication is adjusted based on antimicrobial sensitivity results, with colistimethate sodium or polymyxin B commonly used for drug-resistant organisms. In addition to the application of antibiotics to resist infection, maintaining smooth CSF drainage is also crucial for the treatment of *A. baumannii* intracranial infection, through which CSF drainage can reduce the bacterial concentration of bacteria in CSF, reduce intracranial pressure, and reduce cerebral edema. De Bonis et al.. suggested that intravenous combined intraventricular injection of polymyxin improved the prognosis of the patients and did not result in significant neurotoxicity [22]. Tigecycline does not readily cross the BBB to reach adequate concentrations in infected lesions [39]. Liverana Lauretti et al.., in a pioneering study, documented that the concurrent intravenous and intraventricular administration of tigecycline in a patient post-transsphenoidal resection of a giant pituitary adenoma, who subsequently developed *Multidrug-resistant A. baumannii*, led to a clinical cure without evident neurological toxicity [11]. According to a related study by Huang Q et al.., high concentrations of tigecycline cause neurotoxicity to the nervous system, and tigecycline is not recommended as the treatment of choice for intracranial infections with *A. baumannii* and can be used in small doses twice a day if necessary [40]. 

### Limitations and future directions

In this study, we analyzed in detail the clinical characteristics and prognostic factors associated with *A. baumannii* intracranial infections in patients at a hospital in China, identified six independent predictors of *A. baumannii* intracranial infections, and constructed the first nomogram model to predict the risk of in-hospital mortality in patients. In addition, previous articles have been almost exclusively case studies, and this article has the largest study sample size to date. However, this study has several limitations: first, it is a single-center retrospective study, which is subject to selection bias and information bias; second, we did not study the effect of the duration of antibiotic administration on the prognosis of patients, as some patients received empirical antibiotic treatment at an outside hospital before admission; third, since this study was retrospective, only routine laboratory data were collected, and no additional data on other blood and CSF acute phase reactants and proinflammatory cytokines were available to better assess the role of inflammation. Fourth, the vast majority of the data we included had EVD, and future our study needs further focus on EVD-related infections. Last, even though our sample size was larger than that of previous studies, there was still a lack of sample size in the internal and external validation of the sample data, which is particularly important for validating nomogram models.

## Conclusion

Post-neurosurgical *A. baumannii* intracranial infection remains a severe clinical condition with considerable mortality and public health burden, exacerbated by increasing antimicrobial resistance. Our study highlights the potential value of prognostic modeling in identifying high-risk patients and guiding individualized clinical management. The nomogram developed in this study may serve as a useful reference for future research and clinical application. Continued data collection and expansion of the study cohort are warranted to enhance the robustness and generalizability of these findings.

## Data Availability

The datasets used and/or analyzed during the current study are available from the corresponding author upon reasonable request.

## Abbreviations

CSF: Cerebrospinal fluid
*A. Baumannii*: *Acinetobacter baumannii*
BBB: Blood-brain barrier
MIC: Minimal inhibitory concentration
EVD: External ventricular drainage
VPS: Ventriculoperitoneal shunt
LD: Lumbar drainage
ROC: Receiver operating characteristic
AUC: Area under the curve
ICU: Intensive care unit
GCS: Glasgow coma scale
mRS: Modified rankin scale
mNGS: Macrogenomic next-generation sequencing
